# The moderation effect of identity exploration and basic psychological needs satisfaction on flourishing of Chinese rural children

**DOI:** 10.1186/s41155-020-00166-5

**Published:** 2021-01-04

**Authors:** Sijia Guo, Chau Kiu Jacky Cheung, Jieyi Hu, Xuan Ning

**Affiliations:** 1 College of Public Administration and Humanities, Dalian Maritime University, Dalian, Liaoning China; 2 Department of Social and Behavioral Sciences, City University of Hong Kong, Hong Kong, China; 3 Jinan University, Guangzhou, China; 4 Ryerson University, Toronto, Canada

**Keywords:** Identity exploration, Basic psychological needs, Flourishing, Rural children

## Abstract

Most studies equate children’s mental health to a state of flourishing, which is a positive feeling and functions in their lives. Identity exploration and the satisfaction of three basic psychological needs are universal and crucial indicators of children’s flourishing. First, according to identity crisis theory, children in the pre-adolescence period begin to explore their own identities, a process which significantly affects their development and flourishing. Meanwhile, self-determination theory points out that the basic psychological needs, namely the needs for autonomy, competence, and relatedness, are essential for children’s development and flourishing in the worldwide. Accordingly, this study examined how identity exploration affects the flourishing of rural children in China, one kind of collectivism cultural contexts, with the interaction effect of identity exploration and basic psychological needs satisfaction. To understand the interaction effect of identity exploration and basic psychological needs satisfaction on rural children’s flourishing, we form a theoretical framework combining identity crisis theory and self-determination theory. Both these two theories emphasize the importance of self in facilitating mental health and the development of functioning. Specifically, identity crisis theory focuses on intrapsychic process, while self-determination theory stresses the influence of the surrounding environment on the individual, which provides a solid foundation for integrating these two theories to explore rural children’s flourishing in China. Accordingly, this study collected 520 left-behind children and 475 other rural children in Liaoning Province in Mainland of China, and used regression analysis to measure the associations among variables. This study found that identity exploration and basic psychological needs satisfaction positively affect rural children’s flourishing respectively, while their interaction effects negatively affect on their flourishing.

## Introduction

Since China’s economic reform in 1978, there have been unprecedented changes in Chinese society on a large scale, such as rapid urbanization and industrialization. As a result, a series of social problems developed. One such social problem is that of left-behind children (LBC), children cared for by non-parent caretakers in their hometown for at least 6 months (Dai & Chu, 2018). According to the latest report from a national survey, there were approximately 6.97 million LBC at the end of 2018 (Ministry of Civil Affairs, 2016). This group has been researched from various angles, including the effects of parents’ migration effects, living conditions, mental health status, academic situation, etc. Moreover, most of empirical studies have concluded that LBC were a vulnerable group and suffered from mental illness, such as depression (e.g., Chan, 2009; Cheng & Sun, 2015; Wang & Mesman, 2015). Nevertheless, these empirical studies overlooked the strengths of rural children (Su, Li, Lin, & Zhu, 2017; Xi, Sun, & Xiao, 2006). In addition, most empirical studies ignored other rural children who also face similar challenges and constraints as LBC, such as the poor education condition (Su et al., 2017). These other rural children need attention as well. Therefore, this study turns the focus to flourishing in rural children in China.

Furthermore, there are manifold potential factors influencing children’s flourishing. Among these important factors, identity exploration, which refers to exploring oneself, is a crucial one (Marcia, 1980). During the process of children’s development, identity exploration is an inevitable mission that directly influences their mental health (Berzonsky, 2011; Marcia, 1980). Nevertheless, few studies have assessed how this manifests in children’s mental health in collectivist cultural contexts. Therefore, this study considers its effect on rural children flourishing. In addition, according to self-determination theory, three basic psychological needs are also essential for the individual to thrive. Specifically, these basic psychological needs are autonomy, competence, and relatedness. These are universal humankind needs, and when they are satisfied, children can facilitate their mental health and functioning (Deci & Ryan, 2000). Hence, this study seeks to clarify the associations between identity exploration and satisfaction of basic psychological needs among rural children.

## Literature review

### Ego identity exploration

An important task during adolescence is the formulation of a sense of identity, and identity development refers to culturally accepted, self-relevant values, and future goals (Waterman, 1985). According to Erikson (1968), identity development begins in pre-adolescence and remains a prominent psychosocial task throughout the adolescent and emerging adult period. This means identity formation is a slow process of ego growth in which the identity of childhood gradually changes to a new identity. Furthermore, Marcia (1966) indicated two underlying processes: identity exploration and identity commitment. Identity exploration is the heart of identity transition, in which individuals consider different identity-related options, such as family roles or dating relationships (Erikson, 1959). That is, identity exploration is the degree to which individuals engage in searching for personal values, beliefs, and goals. Some scholars consider identity exploration as a crisis which is not beneficial for the individual’s development and well-being (e.g., Erikson, 1959; Kidwell, Dunham, Bacho, Pastorino, & Portes, 1995). From this viewpoint, the child who is in identity exploration does not have a clear understanding of self and does not have specific goals about the future, and this is threatening for children to develop their identity. However, others argued that identity exploration is helpful for children’s flourishing, as identity development is a slow process whose status changes over time, meaning children who are in identity exploration status can explore themselves in a deeper way and accomplish better identity commitment (e.g., Berzonsky, 1990; Dunkel, 2000).

### Basic psychological needs satisfaction

According to self-determination theory, there are three basic psychological needs universal for humans to feel satisfaction with life. These are the needs for autonomy, relatedness and competence (Deci & Ryan, 1980, 2000). First, autonomy means that the individual can act and behave of his or her volition. That is, the need for autonomy stresses that one’s actions and behaviors can express his or her values and interests. Next, relatedness refers to the feeling of connection to others, receiving care from others, and caring for others. The need for relatedness emphasizes personal perceptions about integration with other people and into the community. Finally, competence means showing one’s abilities in a social context. Likewise, the need for competence stands for enacting one’s roles effectively. Empirical studies show that the satisfaction of these three basic psychological needs can facilitate individuals’ functioning (Deci & Ryan, 2000). Meanwhile, satisfaction of these three basic needs is connected with positive outcomes, including greater general well-being (Deci & Ryan, 2000). This has received support in several ways, including the adoption of an overall index of three needs and the examination of the contribution of each need. For instance, the study by Sheldon, Ryan, and Reis (1996) found that individuals experienced higher positive affect when they accomplished greater needs fulfillment. In addition, studies have tested the association between basic needs satisfaction and well-being in various domains, and generated similar results. For example, Vansteenkiste et al. (2007) discovered that employees experienced higher self-esteem and overall mental health when their basic needs were satisfied in working settings. Meanwhile, studies from other domains, such as the education, found a positive relationship between basic needs satisfaction and overall well-being (e.g., Kasser & Ryan, 1999; Niemiec & Ryan, 2009).

### Flourishing

Childhood and adolescence are periods of intensive development, and successful development during this period has implications for adult development and health. Thus, in the academic field, the mental health of children and adolescents has been of keen interest for researchers, and most studies define mental health as a state of flourishing. As a form of subjective well-being, flourishing refers to the way that life goes well by integrating positive feelings and functioning (Huppert & So, 2013). First, positive feeling refers to the individual’s perceptions and assessment of life, including happiness and satisfaction. Additionally, more than emotional states, flourishing concerns personal functioning in life including positive psychological functioning and positive social functioning (Keyes, 2002). Positive psychological functioning consists of six dimensions to assess personal psychological fulfillment: self-acceptance, positive relationship with others, personal growth, purpose in life, environmental mastery, and autonomy (Ryff, 1989). That is, functioning means that individuals liking themselves for the most part have trusting relationships, make the effort to develop and improve, have an explicit direction in life, make the surrounding environment to satisfy their needs, and have a degree of self-determination. Besides psychological functioning, confronting social challenges and tasks is germane to social well-being (Keyes, 1998). There are five dimensions of social well-being consisting of social coherence, social actualization, social integration, social acceptance, and social contribution. Likewise, individuals function well when they regard society as meaningful, when they believe that society possesses the potential for growth, when they feel they are a part of society, when they accept most parts of society, and when they realize that they can contribute to society. Overall, flourishing concerns personal mental health by combining emotional states and personal functioning from both private and public aspects.

### Theoretical framework

Firstly, Erikson (1963, 1968) proposed identity crisis theory, highlighting the importance of one’s ego identity formation process in the development of personality from childhood into adulthood (Syed & McLean, 2017). From Erikson’s viewpoint, ego identity exploration is a natural and central process during children’s development, and it plays a predominant role in terms of children’s thriving (Darling-Fisher, 2019). Therefore, burgeoning studies adopt the identity crisis perspective to explore the effect of ego identity exploration on children’s mental health and functioning (e.g., Luyckx, Vansteenkiste, Goossens, & Duriez, 2009; Markovitch, Luyckx, Klimstra, Abramson, & Knafo-Noam, 2017; Pellerone, Ramaci, & MiccichÈ, 2018). Additionally, identity crisis theory also assimilates social and cultural indicators when considering personal identity development (Dunkel & Harbke, 2017). However, most studies applying identity crisis theory concentrated on identity issues in Western countries to explore how it manifests in children and adolescents. In other words, there were no substantial studies measuring how identity crisis theory manifests in Eastern culture, as the collectivistic orientation in Eastern culture regards the self differently from the individualistic orientation in Western culture (Oyserman, Coon, & Kemmelmeier, 2002). Moreover, as previously mentioned, the findings about the effects of ego identity exploration on children’s flourishing are still controversial (e.g., Markovitch et al., 2017; Stegarud, Solheim, Karlsen, & Kroger, 1999). Herein, this study adopts identity crisis theory to explore how identity exploration influences flourishing in rural children who are significantly affected by traditional Chinese culture.

Furthermore, self-determination theory proposes that the nature of human beings is to pursue wellness and improve their capabilities, and at the same time, self-determination theory also underlines the importance of context (Adams, Little, & Ryan, 2017; Deci & Ryan, 2008). Specifically, self-determination theory conceives of three universal basic psychological needs, and a supportive environment is conducive to satisfying these needs that optimize the individual (e.g., Hui & Tsang, 2012; Ryan, Bernstein, & Brown, 2010; Ryan & Deci, 2000a). Thereof, self-determination theory emphasizes the importance of sociocultural context, and many researchers also argue that the varied cultural contexts could influence one’s satisfaction of basic psychological needs (e.g., Church et al., 2013; Lekes, Gingras, Philippe, Koestner, & Fang, 2010). This study applies self-determination theory to examine how satisfaction of basic psychological needs influences rural children’s flourishing.

**Fig. 1:**
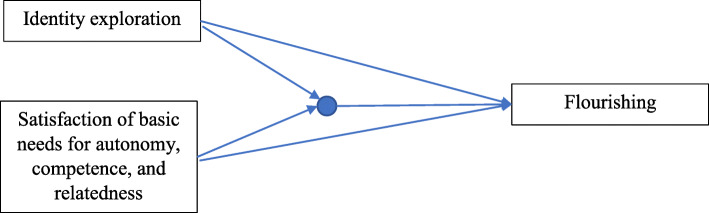
Theoretical framework

In accordance with the nature of the identity crisis and self-determination theories, this study aims to integrate these two theories. First, these two theories focus on individuals’ well-being and functioning development (Erikson, 1968; Schultheiss & Blustein, 1994). Identity crisis theory posits that individuals will promote their well-being if they undergo a well-balanced identity formation process (Luyckx et al., 2009). Likewise, self-determination theory argues that the inner growth is human nature, such that the individual continuously endeavors to live and function well (Deci & Ryan, 2000). Additionally, both theories stress the importance of self during the process of development. Identity crisis theory reasons that children explore themselves and become aware of their uniqueness during the ego identity exploration process, which directly influences their well-being and functioning, and self-determination theory postulates that the individual strives for satisfaction of the basic psychological needs to facilitate one’s well-being and optimize one’s functioning (Deci & Ryan, 2000; Erikson, 1968). Thus, this study integrates these two theories to explore how the ego identity process, satisfaction of basic psychological needs, and their combination influence rural children’s flourishing. Moreover, both identity crisis theory and self-determination theory stress the influence of cultural context; therefore, this study also considers the effect of traditional Chinese culture as an antecedent. Herein, the hypotheses are as follows regarding the rural child in China (see Fig. 1): H1: Ego identity exploration positively affect on flourishing. H2: Satisfaction of basic psychological needs positively affect on flourishing. H3: The combination of ego identity exploration and satisfaction of basic psychological needs positively affect on flourishing.

## Methodology

### Samples and procedure

This study collected 1002 rural children through a self-reported survey including both LBC and other rural children in Liaoning Province at the end of 2017. Meanwhile, this study adopted a school-based convenience sampling approach to recruit research participants. At the beginning, the procedure selected three different cities with various economic levels, then six towns within the selected cities. Next, the procedure selected four primary schools and three junior high schools within the six towns. Then, the sampling proceeded with the use of the student roster for random selection of children, consisting of LBC and other rural children in each of the selected schools. Following this procedure, the data from 520 LBC and 475 other rural children (non-LBC) were valid.

### Measurements

#### Left-behind status

Due to the characteristics of rural China, there are two major groups of rural children: LBC and other rural children. Considering this specificity, this study used several questions to separate the children into two groups. “Who has been your caretaker in the past 6 months?” “Have you changed caretakers in the past six months?” “With whom have you lived in the past 6 months?” The child would be regarded as left behind if both of resident and caretaker were not his or her a parent. Through these questions, this study differentiated between LBC and other rural children.

#### Flourishing

The Mental Health Continuum-Short Form Scale (MHC-SF) was adapted to measure children’s flourishing status, which includes 14-item (Guo et al., 2015; Keyes, 2005). There are three dimensions of this scale that are hedonic well-being (3 items), personal functioning (6 items) and social functioning (5 items) respectively. Meanwhile, respondents assessed their feelings in the past week on a 6-point scale. Higher scores indicated higher flourishing. The reliability coefficient of this scale was .895.

#### Traditional Confucian values

Traditional Chinese culture, particularly Confucianism, profoundly influences Chinese behaviors, goals, and values (Nelson & Chen, 2007). Therefore, this study adopted the Traditional Confucian Value Scale (TCVS) to measure the people’s value in the living context (Yang, 1993). There were 40 items that were simple and direct phrases, for instance, hardworking, self-disciplined, and loyal to family. Each respondent rated his or her perception of the value in context on a 4-point scale in past six months. High scores implied that the individual held the values of Confucianism strongly. The reliability coefficient of this scale was .901.

#### Basic needs satisfaction

The Basic Psychological Needs Satisfaction Scale for Children (BPNS-C) (Chen et al., 2015) was adapted to measure basic needs satisfaction. This scale measured the following dimension of basic psychological needs in general in last 1 month.

#### Autonomy

Autonomy refers to the perception of being oneself, and the need for autonomy represents a sense of behaving and acting in accordance to personal volition and values. Eight questions measured autonomy. A sample question was “I feel a sense of choice and freedom in the things I undertake.” Each respondent rated his or her perception in the past one month through a 5-point rating scale, of which a higher score indicated higher autonomy. The reliability coefficient of this sub-scale was .635.

#### Competence

Competence means the sense of efficacy in dealing with daily life. Likewise, eight questions examined competence. Sample questions were “I can do things well”, “I am good at what I do”, and “I can achieve my goals.” At the same time, each respondent rated the experience on a 5-point rating scale, of which a higher score indicated higher satisfaction of the need for competence. The reliability coefficient of this sub-scale was .749.

#### Relatedness

Relatedness signifies the feeling of being related to and cared about by others. Eight questions measured the need for relatedness as well. Sample questions were “The people that I like, also like me”, “I feel close to the people I care about,” and “I feel close to and connected with the people who are important to me.” Moreover, each respondent rated his or her feeling on a 5-point scale. Higher scores indicated higher satisfaction of the need for relatedness. The reliability coefficient of this sub-scale was .728.

#### Satisfaction of basic psychological needs

Overall, satisfaction of basic psychological needs was the composite of satisfaction of the needs for autonomy, competence, and relatedness. The reliability coefficient of this overall scale was .900.

#### Identity exploration

This study adopted the Ego Identity Process Exploration (EIPE) (Berman, Weems, & Stickle, 2006) to measure the identity exploration of LBC and non-LBC. The original scale covers the attitudes toward religion, politics, dating relationships, friendship, occupation, and the role in the family. However, according to the reality of the Chinese context, this study modified the original one by deleting the questions about religion and politics. Herein, there were 12 items to assess the identity exploration status of LBC and non-LBC. Sample questions were “My values are likely to change in the future”, “I have re-examined many different values in order to find the ones that are best for me.” In the same way, each respondent rated his or her experience on a 5-point rating scale in last six months. A higher score indicated a higher level of identity exploration. The reliability coefficient of this sub-scale was .694.

#### Sociodemographic variables

The sociodemographic variables in this study included gender, age, and socioeconomic status (SES). The female children scored 1, while the male scored 0. Age was measured in years, and the average age was 13 years. SES was composed of the educational level of parents and caretakers, along with the yearly household income. Regarding educational level, seven categories were from “never attached to school” to “university graduate or above,” and most of parents and caretakers had attained the senior high school level of education. Furthermore, the yearly household income was indicated by lower than 100,000 RMB, between 100,000 RMB and 120,000 RBM, higher than 120,000 RMB, and do not know. As such, 64.7% participant did not report yearly household income whereas 11.1% of participants and 12.6% of participants indicated that their yearly household income was above 120,000 and below 100,000 respectively.

### Data analysis

Regression analysis held flourishing as the outcome and basic needs satisfaction, identity-exploration, their interactions and backgrounds as predictors. Notably, the interaction terms were products based on standard scores to minimize problems due to multicollinearity. The analysis hierarchically included background characteristics as control variables, and identity-exploration in the first block, basic needs satisfaction’s components and overall basic needs satisfaction in the second block, and the interaction terms in the third block.

## Results

### Effects of basic needs satisfaction and its sub-components on flourishing of rural children

In the very beginning, when overall basic needs satisfaction was the only predictor, it indicated a significant positive effect on flourishing (*β* = .448, *p* < .01). Moreover, after controlling other background characteristics, although the effect of overall basic needs satisfaction decreased, it also influenced flourishing in a positive way (*β* = .340, *p* < .001, see Table 1). Meanwhile, the effects of the caretaker’s educational level (*β* = .118, *p* < .01) and traditional Chinese culture (*β* = .278, *p* < .001) were significantly positive. On the contrary, the effect of left-behind status (*β* = − .060, *p* < .05) was significantly negative.

**Table 1:** Main effects on flourishing

Predictor	Model 1	Model 2	Model 3	Model 4	Model 5
Left-behind status	− .060*	− .064*	− .055	− .059*	− .052
Age	− .043	− .043	− .028	− .045	− .042
Female	.030	.032	.059*	.033	.081**
Yearly household income	− .009	− .009	− .005	− .015	− .009
Caretaker’s education level	.118**	.124***	.111*	.122**	.106**
Mother’s education level	.009	.005	.015	.006	.012
Father’s education level	− .031	− .032	− .026	− .019	− .011
Traditional Chinese culture	.278***	.315***	.332***	.316***	.376***
Ego identity exploration					.142***
Overall basic psychological needs	.340***				
Autonomy		.306***			
Competence			.251***		
Relatedness				.248***	
Constant	-.215	.051	.170	.478	.708

***p < .001, **p < .01, *p < .05. *R*^*2*^_*1*_ *= .293*, *R*^*2*^_*2*_ *= .279*, *R*^*2*^_*3*_ *= .252*, *R*^*2*^_*4*_ *= .247*, *R*^*2*^_*5*_ *= .213*

Additionally, when each basic psychological need became the only predictor, the results showed that autonomy (*β* = .399, *p* < .001), competence (*β* = .351, *p* < .001), and relatedness (*β* = .360, *p* < .001) were significantly conducive to flourishing (see Table 1). Moreover, when the needs of autonomy and relatedness were the predictor, after the control for background characteristics, the effects of the caretaker’s educational level, left-behind status and traditional Chinese culture maintained. However, when competence was the predictor, the effect of LBC was not significant, while girls exhibited higher flourishing than did boys. In brief, as postulated, every basic psychological need, especially autonomy, had a strong positive effect on rural children’s flourishing.

### Effects of ego identity exploration on flourishing of rural children

As the only predictor, ego identity exploration had a significant positive effect on flourishing (*β* = .197, *p* < .001). However, after the control for background characteristics, the effect of identity-exploration decreased (*β* = .142, *p* < .001, see Table 1). Meanwhile, the effects of caretaker’s educational level (*β* = .106, *p* < .01) and traditional Chinese culture in the living context (*β* = .376, *p* < .001) on flourishing were strongly significantly positive. This indicates that if rural children explore their identity as a deep process, they will experience higher flourishing.

### Interaction effects of overall basic needs satisfaction and its sub-components with identity exploration on flourishing of rural children

The interaction effect of overall basic needs satisfaction and identity exploration (*β* = − .093, *p* < .001, see Table 2) was significantly negative regarding flourishing. Likewise, the interaction effect of autonomy and ego identity exploration (*β* = − .095, *p* < .001), competence and ego identity exploration (*β* = − .079, *p* < .01), and relatedness and ego identity exploration (*β* = − .085, *p* < .01) were significantly negative regarding flourishing respectively. The effects meant that overall basic needs satisfaction and its sub-components were in competition with ego identity exploration. Obviously, the interaction effect of autonomy and identity exploration is the greatest but is negative. Overall, the effects of overall basic needs satisfaction and its sub-components were redundant and overlapped with the effect of identity exploration on flourishing. That is the effects were competing with rather than complementing to each other.

**Table 2:** Main and interaction effects on flourishing

Predictor	Model 1	Model 2	Model 3	Model 4
Left-behind status	− .048	− .055*	− .042	− .050
Age	− .054*	− .054	− .036	− .061*
Female	.047	.044	.075**	.052
Yearly household income	− .006	− .008	− .002	− .012
Caretaker’s education level	.106**	.113**	.099**	.109**
Mother’s education level	.009	.004	.016	.008
Father’s education level	− .027	− .027	− .023	− .016
Traditional Chinese culture	.252***	.289***	.309***	.287***
Ego identity exploration	.160***	.152***	.148***	.164***
Overall needs satisfaction	.343***			
Ego identity exploration* Overall needs satisfaction	-.093***			
Autonomy		.314***		
Ego identity exploration* Autonomy		− .095***		
Competence			.249***	
Ego identity exploration *Competence			− .079**	
Relatedness				.260***
Ego identity exploration *Relatedness				− .085**
Constant	− .687	− .403	− .263	.007

***p < .001, **p < .01, *p < .05. R^*2*^_*1*_ *= .323*, *R*^*2*^_*2*_ *= .308*, *R*^*2*^_*4*_ *= 278*

## Discussion

Flourishing is an important indicator for assessing children’s development, and it is essential to explore the potential protective and risk factors for flourishing (Eccles, 1999; Eccles, Brown, & Templeton, 2008). Therefore, we investigated how identity exploration and basic psychological needs satisfaction influence flourishing by integrating identity crisis theory and self-determination theory. We have tried to understand flourishing of rural children better and give suggestions for their development.With respect to ego identity development predicated on identity crisis theory, identity exploration and commitment are two dimensions to help define the adolescent’s identity (Luyckx et al., 2007). Exploration refers to the conscious deliberation of alternative goals and roles, while commitment is the formation of these deliberations (Marcia, 1966). Theoretically speaking, most people would experience different identity statuses and an identity crisis before accomplishing commitment. If individuals constantly suffer an identity crisis, they will experience emotional issues and deviant behaviors, such as anxiety (e.g., Côté & Schwartz, 2002; Dumas, Ellis, & Wolfe, 2012). However, before or in early adolescence, it is natural to be in exploration status. Accordingly, since all participants in our study are primary school and first-year secondary school students, they have not reached the stage of identity commitment, and their identity exploration is crucial for their future identity development. In support of this observation, this study finds that identity exploration maintained a positive effect on flourishing. All participants are in the pre-adolescence period, and exploration means that they can explore their alternative values, beliefs, and identities intensely to help them develop positive emotions and functioning to increase their flourishing. This result is consistent with the prior hypothesis. Meanwhile, the study from Berzonsky (1992) supported the idea that proactive identity exploration helps individuals to engage in the process by seeking and evaluating self-relevant information. Furthermore, the process of identity exploration encourages individuals to be self-critical and constantly revise their identities by receiving information (Luyckx et al., 2008). In addition, Erikson (1968) also proposed that identity formation is a function of psychosocial factors. That is, apart from inherently development, ego identity is also constructed within in a social context. From this viewpoint, this study finds that identity exploration plays a positive role in pre-adolescents’ flourishing. This means that the pre-adolescents can improve their emotional status and enhance their functioning in psychological and social perspectives in collectivistic orientations. In a word, identity exploration is beneficial for rural children’s flourishing.

Basic psychological needs satisfaction was a contributor to flourishing in accordance with self-determination theory (Luyckx et al., 2009). Here, we find that the overall basic psychological needs satisfaction and its three basic components (i.e., competence, autonomy and relatedness) contribute to flourishing. This finding corresponds to existing studies showing that satisfaction of these basic psychological needs are the foundations of personal growth and well-being (e.g., Sheldon et al., 1996; Weinstein & Ryan, 2010). First, self-determination theory postulates that the human is naturally inclined to learn, grow, assimilate cultural values, and to connect to others (Deci & Ryan, 1985). Self-determination theory does not overstate the positive perspectives of human beings, but rather puts forward the idea that people can behave in bad ways, such as acting selfishness, which is the outcome of need-frustrating conditions. In other words, self-determination theory links basic needs satisfaction with the feelings of wellness. Thereby, satisfaction of the basic psychological needs leads people to assess optimal functioning and to enhance their well-being (Weinstein & Ryan, 2010). Our findings also support this through verifying that satisfaction of overall basic psychological needs fosters the flourishing of rural children. Additionally, self-determination theory suggests that there are three basic components involved in basic psychological needs: supports for autonomy, competence, and relatedness, which enable people to be active, to thrive, and to function effectively in multiple domains (e.g. Baard, Deci, & Ryan, 2004; Ryan et al., 2010). Furthermore, autonomy particularly counts as the core of needs fulfillment and thriving. When people can behave autonomously, it means that people can choose and develop themselves in their preferred ways to actuate well-being (Ryan & Deci, 2011). The results in this study are in favor of the arguments that satisfaction of all these basic psychological needs can facilitate rural children’s flourishing. Notably, the satisfaction of the need for autonomy need has the most significant influence.

This study proposes the interaction effects of basic needs satisfaction and identity exploration on flourishing, and we found that identity exploration, overall basic psychological needs satisfaction, and its sub-components (i.e., competence, autonomy, and relatedness) have positive effects on flourishing, while their conjunctions have a negative interaction effect on flourishing. There are four possible explanations for these negative interaction effects. First, during the ego identity exploration process, children are continuously looking for diverse alternatives in terms of their goals, values, and convictions in identity, without establishing a committed self (Luyckx, Soenens, Goossens, Beckx, & Wouters, 2008; Marcia, 1966). However, according to self-determination theory, the individual with explicit life goals is more likely to satisfy the inborn psychological needs, namely autonomy, competence and relatedness (Deci & Ryan, 2008). From this perspective, there is a conflict between ego identity exploration and basic psychological needs satisfaction. Next, the need for autonomy highlights the self and volition, and this is consistent with the nature of ego identity exploration, which stresses individualism. Nevertheless, in the context of traditional Chinese culture, collectivism predominantly emphasizes the community and group (Hsu & Huang, 2016). Therefore, ego identity exploration and autonomy are redundant in the rural Chinese cultural context.

In addition, during the ego identity exploration process, the individual increasingly enhances abilities and competencies, such as critical thinking and choosing, which help the individual explore his or her similarities and differences in comparison with others (Erikson, 1968). Likewise, the need for competence requires individuals to strengthen their capabilities to master the environment (Ryan & Deci, 2000b). Thus, ego identity exploration and the need for competence overlap in emphasizing the individual’s capabilities and abilities. As such, to promote one’s perception of competence, the child needs to put more effort in to make progress, and this likely hurts the child’s flourishing.

Finally, ego identity exploration is a process to explore one’s existence in the wake of intrapsychic separation (Kroger, 2000). During this process, the individual tends to be independent and autonomous, while the need for relatedness refers to one’s perception of belongingness. Therefore, the nature between ego identity exploration and the need for relatedness is contradictory to some extent.

To sum up, due to the overlap and contradiction between ego identity exploration and basic psychological needs, their interaction effects were negative on rural children’s flourishing.

Beyond that, there are other factors influencing rural children’s flourishing. First, it is apparent that traditional Chinese culture has a very strong effect on their flourishing. Traditional Chinese culture, namely Confucianism, is rooted in rural China, and it profoundly influences rural children’s thoughts and behaviors (Hsu & Huang, 2016; Nelson & Chen, 2007). Traditional Chinese culture highlights the significance of humanism, self-improvement, harmonious relationships and one’s achievement, which correspond to the essence of flourishing (Hui & Tsang, 2012). Herein, the values derived from traditional Chinese culture sustain rural children’s flourishing. In the same vein, some empirical studies have verified the positive effect of traditional Chinese culture (e.g., Hui & Tsang, 2012; Zeng & Guo, 2012) For instance, Lu and Gilmour (2004) demonstrated that the cultural context indeed affected individuals’ flourishing. Traditional Chinese culture stressed the cultivation of one’s spirit, which positively influenced Chinese’ flourishing. Overall, people in different cultures have varied comprehension and perceptions regarding flourishing, and this study finds that traditional Chinese culture fosters rural children’s flourishing substantially.

Additionally, this study also finds that LBC are more likely to experience lower flourishing than other rural children although the differences are small. This result is consistent with most empirical studies (e.g., Chan, 2009; Wang & Mesman, 2015). Due to parental separation, LBC lack adequate physical company and emotional support, and these are detrimental to their flourishing. However, left-behind status is not decisive, and the negative effect of left-behind status could decrease or disappear after the control for other protective factors. In contrast, this study finds that caretakers’ education promotes rural children’s flourishing. Parental investment, of which parental education is one facet, is essential for the child’s well-being and development (Sayer, Gauthier, & Furstenberg Jr, 2004). In other words, parents with higher education can better invest in their children’s well-being (Coleman, 1988). In this study, caretakers consist of those looking after LBC in daily life and the parents of other rural children. Thereof, caretakers’ education is an important protective factor of rural children’s flourishing.

The implications of this study unfold in two perspectives. First, integrating identity crisis theory and self-determination theory helps explain flourishing in Chinese rural children. Consequently, there are overlapping items in both two theories, which thus generate a negative interaction effect on flourishing. Moreover, this study also examines the idea that in the pre- or middle-adolescence period, exploring an identity has a positive effect on flourishing. At the same time, from a practical perspective, results from this study suggest that it is crucial to pay attention to all rural children rather than only emphasizing LBC. Furthermore, the government, the community and schools also need to heed adolescents’ psychosocial development. This study sheds light on the experience of rural children in China, showing the empirical features of basic needs satisfaction and identity exploration. However, there are several limitations that still need to be addressed. First, self-report data are subjective and at risk of inaccuracy. Therefore, more measures to help to collect accurate data are required. In addition, all participants are from three cities from China’s Liaoning Province; hence, this study best showed the situation in these cities. More cities in China should be included in future research. What is more, the cross-sectional design in this study hinders causal certainly in the effects of identity exploration and basic needs satisfaction on flourishing. A panel research design is highly recommended for future research.

## Conclusions

This study delineates the importance of rural adolescents’ flourishing. In terms of the contributions, this study integrates identity crisis and self-determination theories to explore and elaborate on adolescents’ flourishing in a rural Chinese context, and this study found a negative interaction effect between ego identity exploration and basic psychological needs satisfaction. In other words, overemphasizing self is not conducive to rural adolescents’ flourishing. In addition, in accordance with the empirical findings, this study also has practical implications. In rural China, both traditional Chinese values and values from Western culture influence adolescents due to urbanization and industrialization. Facing the coexistence of two values, it is necessary for government, community, and school to cooperate with each other to focus on the psychosocial development and to foster the flourishing of rural adolescents. For instance, the community and school can organize activities for rural adolescents and encourage them to explore their interests and strengths. Overall, this study complements the empirical studies on rural adolescents, and the findings of this study enrich the knowledge of identity crisis and self-determination theories.

## Data Availability

None

## Abbreviations

LBC: Left-behind children
MHC-SF: The Mental Health Continuum-Short Form Scale
TCVS: The Traditional Confucian Value Scale
BPNS-C: The Basic Psychological Needs Satisfaction Scale for Children
*β*: Standardized coefficient
